# Supplementary material for: The Chloroplast Envelope of Angiosperms Contains a Peptidoglycan Layer
Source: Cells. 2023 Feb 9;12(4):563. doi: 10.3390/cells12040563 (PMC9954125; doi:10.3390/cells12040563)
Supplement: Supplementary file 1 [file cells-12-00563-s001.zip › cells-2210480-supplementary.pdf]

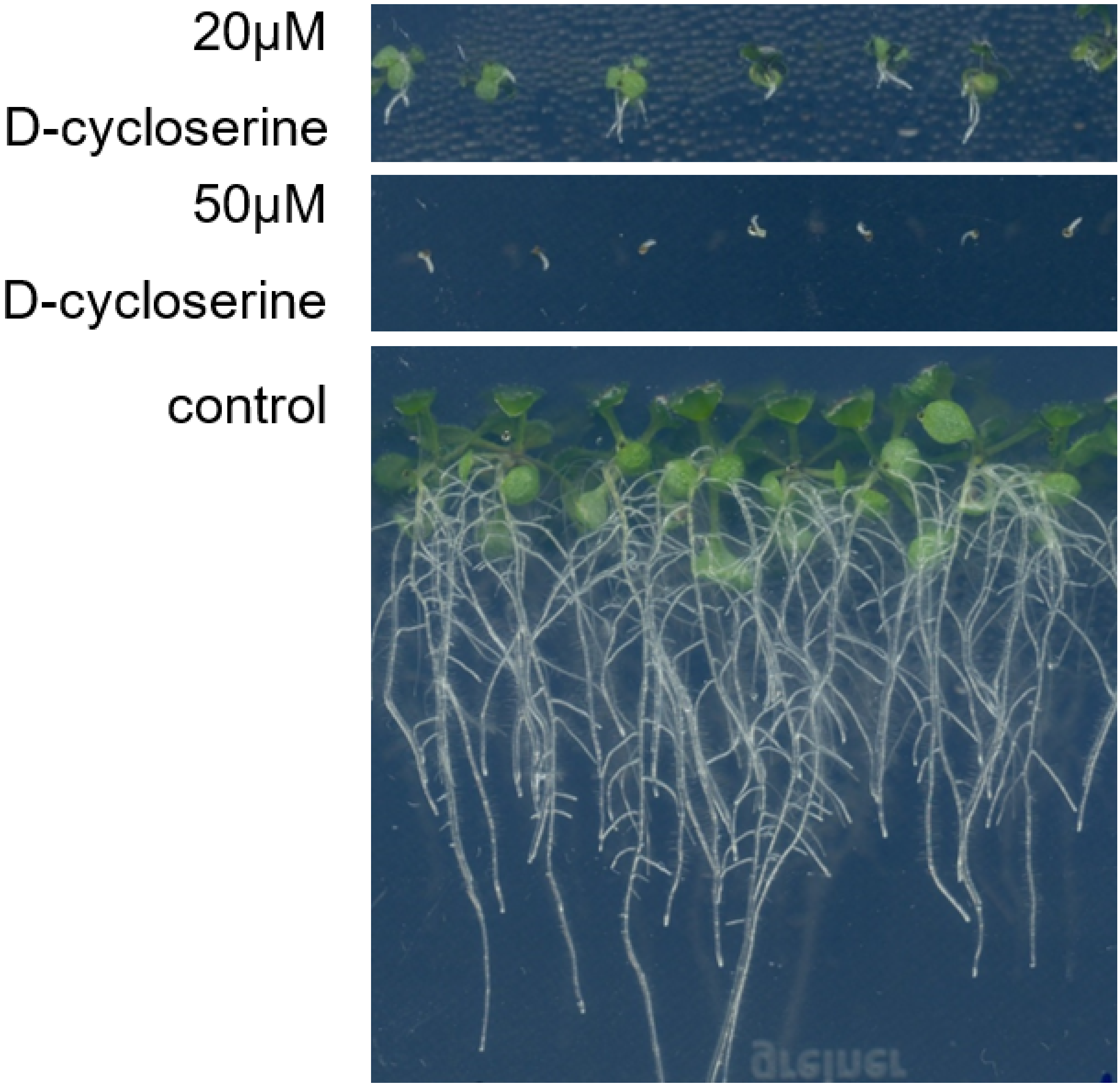

Figure S1. Arabidopsis seedling germination is inhibited by D-cycloserine. Arabidopsis seeds (Col-0) were germinated for 10 days under long day conditions on growth media containing 0, 20 and 50 μM D-cycloserine.

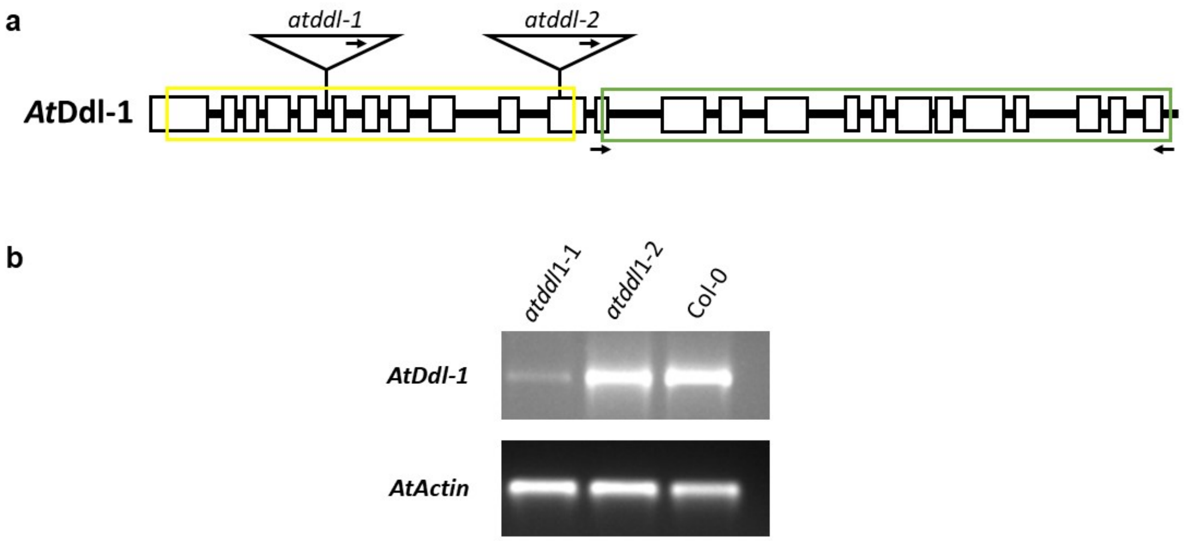

Figure S2. Transcription of *At-DDL* in *atddl-1* and *atddl-2.* (**a**) Structure of the At-DDL gene. Exons and introns are represented by black boxes and lines, respectively. Positions of insertion sites of *atddl-1* and *atddl-2* are marked by triangles. Arrows in the triangles indicate position of left border in the insertions. Genomic regions encoding putative complete DDL enzymes are framed with yellow and green boxes, respectively. Arrows under the scheme mark the positions of the primers used for RT-PCR analysis. (**b**) RT-PCR analysis of *AtDDL-1* in *atddl-1*, *atddl-2* and corresponding wild type seedlings (Col-0). Expression of *AtDDL-1* is shown in the upper row, whereas expression of constitutive control (*AtActin2*) is shown below.

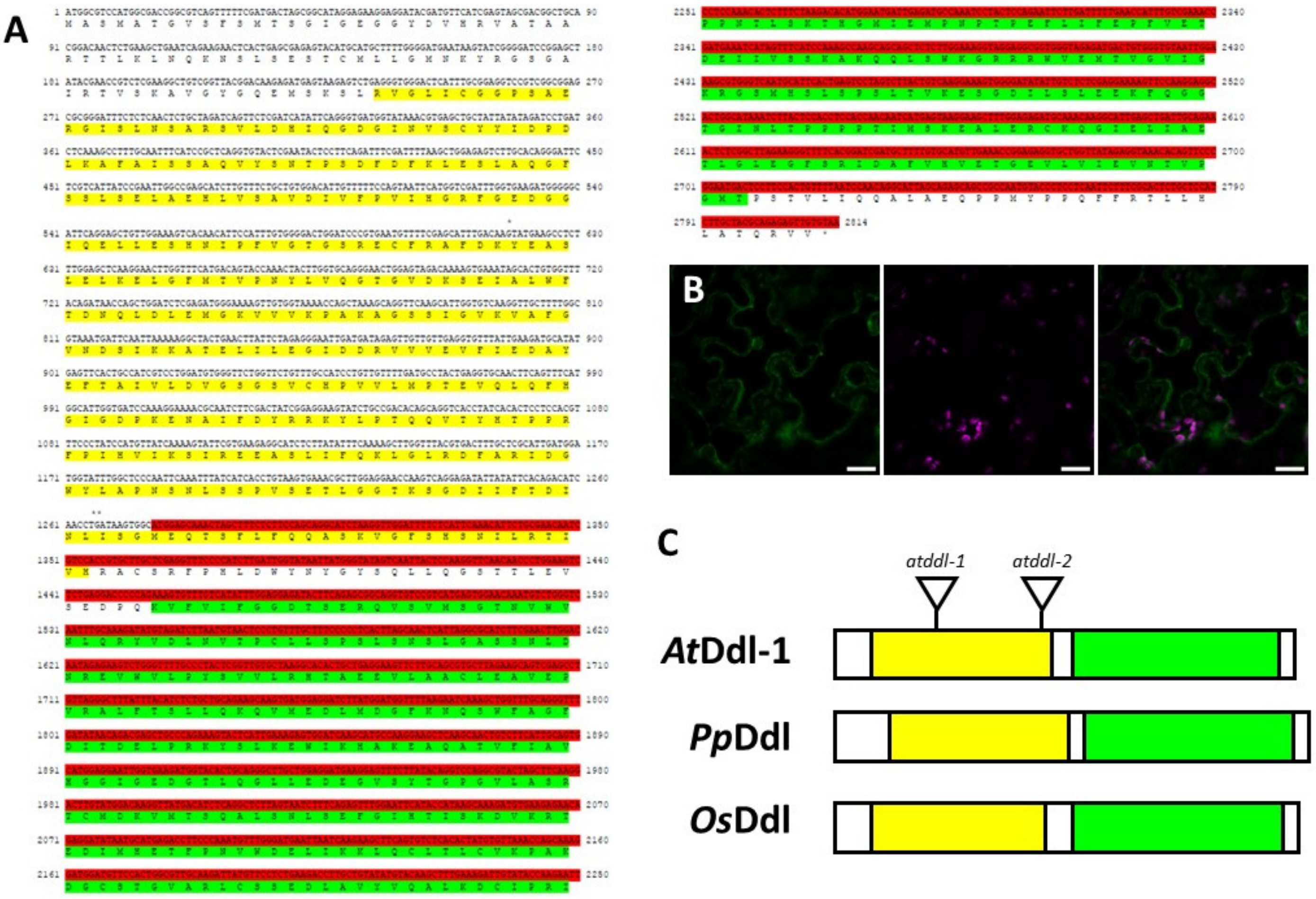

Figure S3. *At-DDL*ΔN-Term encodes for a translatable putative DDL enzyme. (**A**) Coding sequence of *At-DDL1.2*. The peptide sequence of the putative N-terminal DDL enzyme is highlighted in yellow, the putative C-terminal DDL enzyme is highlighted in green. The coding sequence of *At-DDL*ΔN-Term is highlighted in red. The T-DNA insertion sites (on cDNA level) of *ddl-1* and *ddl-2* are marked by one and two asterisks, respectively. (**B**) Fluorescence microscopic image of *Nicotiana* cells expressing *At-DDL*ΔN-Term-GFP (green, left), image of chlorophyll autofluorescence (magenta, middle) and merge of both images (right). (**C**) Schematic representation of the DDL protein structure of *Arabidopsis thaliana* (*At*DDL-1), *Physcomitrella patens* (*Pp*DDL, BAE45860), and *Oryza sativa* (*Os*DDL, BAF22630). The protein regions of the putative N- and C-terminal DDL enzymes are highlighted in yellow and green, respectively. Additionally, the insertion sites of T-DNAs in *atddl-1* and *atddl-2* are marked by triangles.

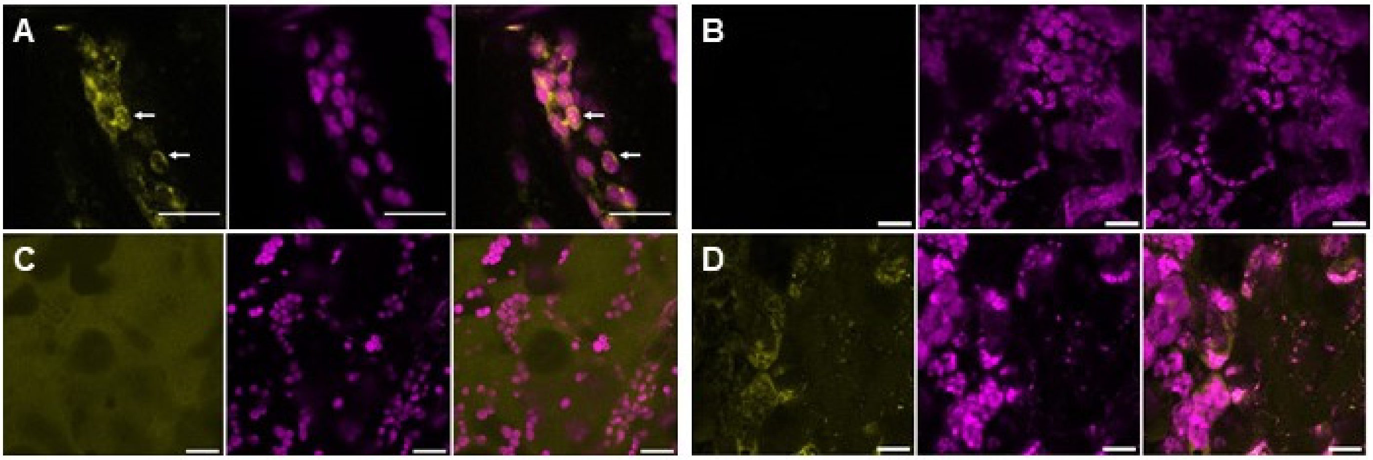

Figure S4. Control experiments for the detection of PGN in higher plants via click chemistry. (**A**) Fluorescent detection of PGN around plastids in *Physcomitrella patens* protonema cells after click chemistry reactions of ATTO 514-Alkyne with ADA (scale bar 10 µm). (**B**) Fluorescence microscopic images of *Arabidopsis* Col-0 seedlings incubated with a click chemistry reaction mix only with ADA (scale bar 20 µm). (**C**) Fluorescence microscopic images of *Arabidopsis* Col-0 seedlings incubated with a click chemistry reaction mix only with ATTO514-Alkyne (scale bar 20 µm). (**D**) Fluorescence microscopic images of *Arabidopsis* Col-0 seedlings treated with 10 µM D-cycloserine after click chemistry reactions of ATTO 514-Alkyne with ADA (scale bar 20 µm). Left image ATTO-514 fluorescence, middle image chlorophyll autofluorescence, right image merged image. White arrows indicate chloroplasts surrounded by fluorescent ring-like structures.

DmPGRP$_{\Delta SP}$

```
  1 ATGGGAAAGTCTAGACAAAGATCTCCTGCTAATTGTCCTACTATTAAGCTTAAGAGACAATGGGGAGGAAAGCCTTCTCTTGGACTTCAT 90
    M  G  K  S  R  Q  R  S  P  A  N  C  P  T  I  K  L  K  R  Q  W  G  G  K  P  S  L  G  L  H

 91 TATCAAGTTAGACCTATTAGATATGTTGTTATTCATCATACTGTTACTGGAGAATGTTCTGGACTTCTTAAGTGTGCTGAAATTCTTCAA 180
    Y  Q  V  R  P  I  R  Y  V  V  I  H  H  T  V  T  G  E  C  S  G  L  L  K  C  A  E  I  L  Q

181 AATATGCAAGCTTATCATCAAAATGAACTTGATTTTAATGATATTTCTTATAATTTTCTTATTGGAAATGATGGAATTGTTTATGAAGGA 270
    N  M  Q  A  Y  H  Q  N  E  L  D  F  N  D  I  S  Y  N  F  L  I  G  N  D  G  I  V  Y  E  G

271 ACTGGATGGGGACTTAGAGGAGCTCATACTTATGGATATAATGCTATTGGAACTGGAATTGCTTTTATTGGAAATTTTGTTGATAAGCTT 360
    T  G  W  G  L  R  G  A  H  T  Y  G  Y  N  A  I  G  T  G  I  A  F  I  G  N  F  V  D  K  L

361 CCTTCTGATGCTGCTCTTCAAGCTGCTAAGGATCTTCTTGCTTGTGGAGTTCAACAAGGAGAACTTTCTGAAGATTATGCTCTTATTGCT 450
    P  S  D  A  A  L  Q  A  A  K  D  L  L  A  C  G  V  Q  Q  G  E  L  S  E  D  Y  A  L  I  A

451 GGATCTCAAGTTATTTCTACTCAATCTCCTGGACTTACTCTTTATAATGAAATTCAAGAATGGCCTCATTGGCTTTCTAATCCT 534
    G  S  Q  V  I  S  T  Q  S  P  G  L  T  L  Y  N  E  I  Q  E  W  P  H  W  L  S  N  P
```

HsPGRP$_{\Delta SP}$

```
  1 ATGCAAGAAACTGAAGATCCTGCTTGTTGTTCTCCTATTGTTCCTAGAAATGAATGGAAGGCTCTTGCTTCTGAATGTGCTCAACATCTT 90
    M  Q  E  T  E  D  P  A  C  C  S  P  I  V  P  R  N  E  W  K  A  L  A  S  E  C  A  Q  H  L

 91 TCTCTTCCTCTTAGATATGTTGTTGTTTCTCATACTGCTGGATCTTCTTGTAATACTCCTGCTTCTTGTCAACAACAAGCTAGAAATGTT 180
    S  L  P  L  R  Y  V  V  V  S  H  T  A  G  S  S  C  N  T  P  A  S  C  Q  Q  Q  A  R  N  V

181 CAACATTATCATATGAAGACTCTTGGATGGTGTGATGTTGGATATAATTTTCTTATTGGAGAAGATGGACTTGTTTATGAAGGAAGAGGA 270
    Q  H  Y  H  M  K  T  L  G  W  C  D  V  G  Y  N  F  L  I  G  E  D  G  L  V  Y  E  G  R  G

271 TGGAATTTTACTGGAGCTCATTCTGGACATCTTTGGAATCCTATGTCTATTGGAATTTCTTTTATGGGAAATTATATGGATAGAGTTCCT 360
    W  N  F  T  G  A  H  S  G  H  L  W  N  P  M  S  I  G  I  S  F  M  G  N  Y  M  D  R  V  P

361 ACTCCTCAAGCTATTAGAGCTGCTCAAGGACTTCTTGCTTGTGGAGTTGCTCAAGGAGCTCTTAGATCTAATTATGTTCTTAAGGGACAT 450
    T  P  Q  A  I  R  A  A  Q  G  L  L  A  C  G  V  A  Q  G  A  L  R  S  N  Y  V  L  K  G  H

451 AGAGATGTTCAAAGAACTCTTTCTCCTGGAAATCAACTTTATCATCTTATTCAAAATTGGCCTCATTATAGATCTCCT 528
    R  D  V  Q  R  T  L  S  P  G  N  Q  L  Y  H  L  I  Q  N  W  P  H  Y  R  S  P
```

Figure S5. Coding sequences and amino acid sequences of *DmPGRP*$_{\Delta SP}$ and *HsPGRP*$_{\Delta SP}$.

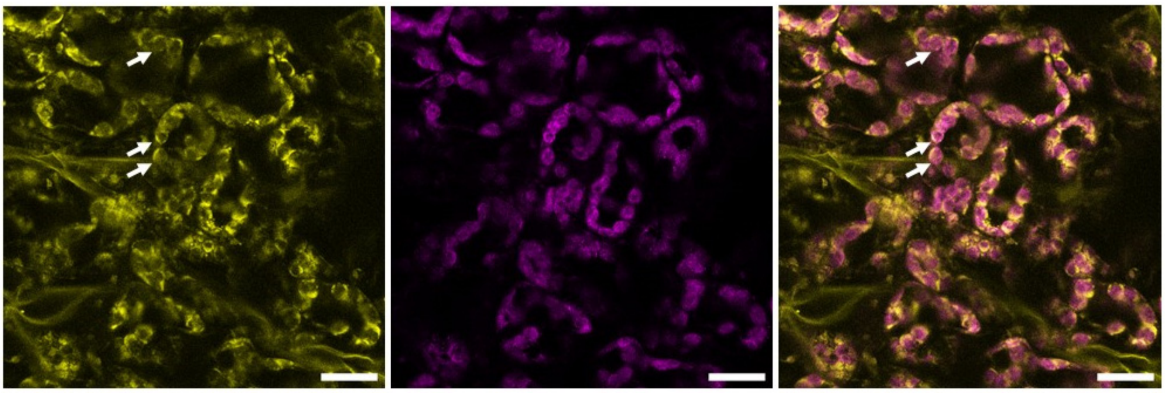

Figure S6. Visualization of PGN around chloroplasts of *atddl-1.* Fluorescent detection of PGN around plastids in *atddl-1* seedling cells after click chemistry reactions of ATTO 514-Alkyne with ADA (scale bar 20 μm). Left image ATTO-514 fluorescence, middle image chlorophyll autofluorescence, right image merged image. White arrows indicate chloroplasts surrounded by fluorescent ring-like structures.

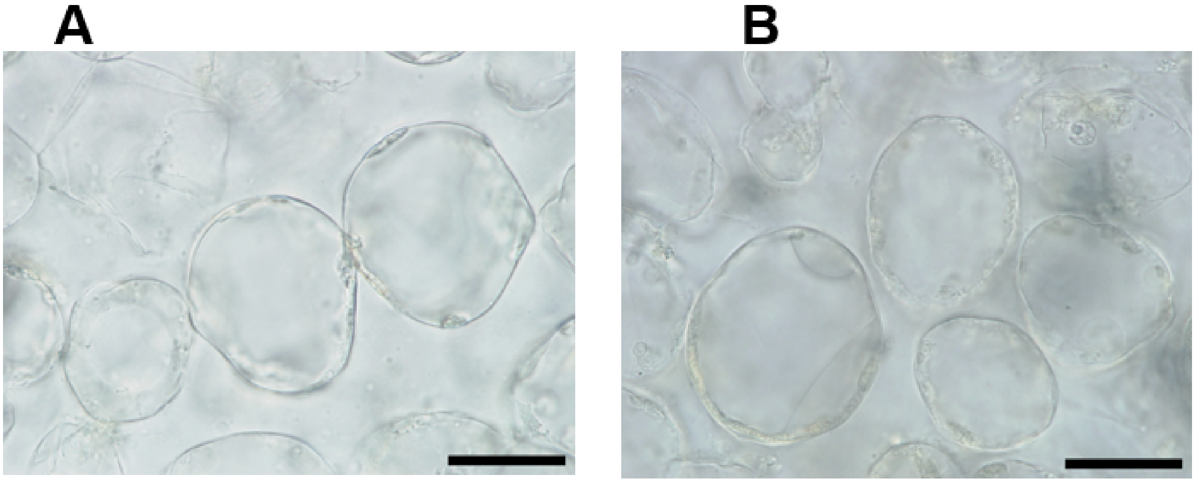

Figure S7. Bright field microscopic images of cotyledon cells from (**A**) *atmurE-3* and (**B**) *atmurE-4* (scale bar 30 μm).

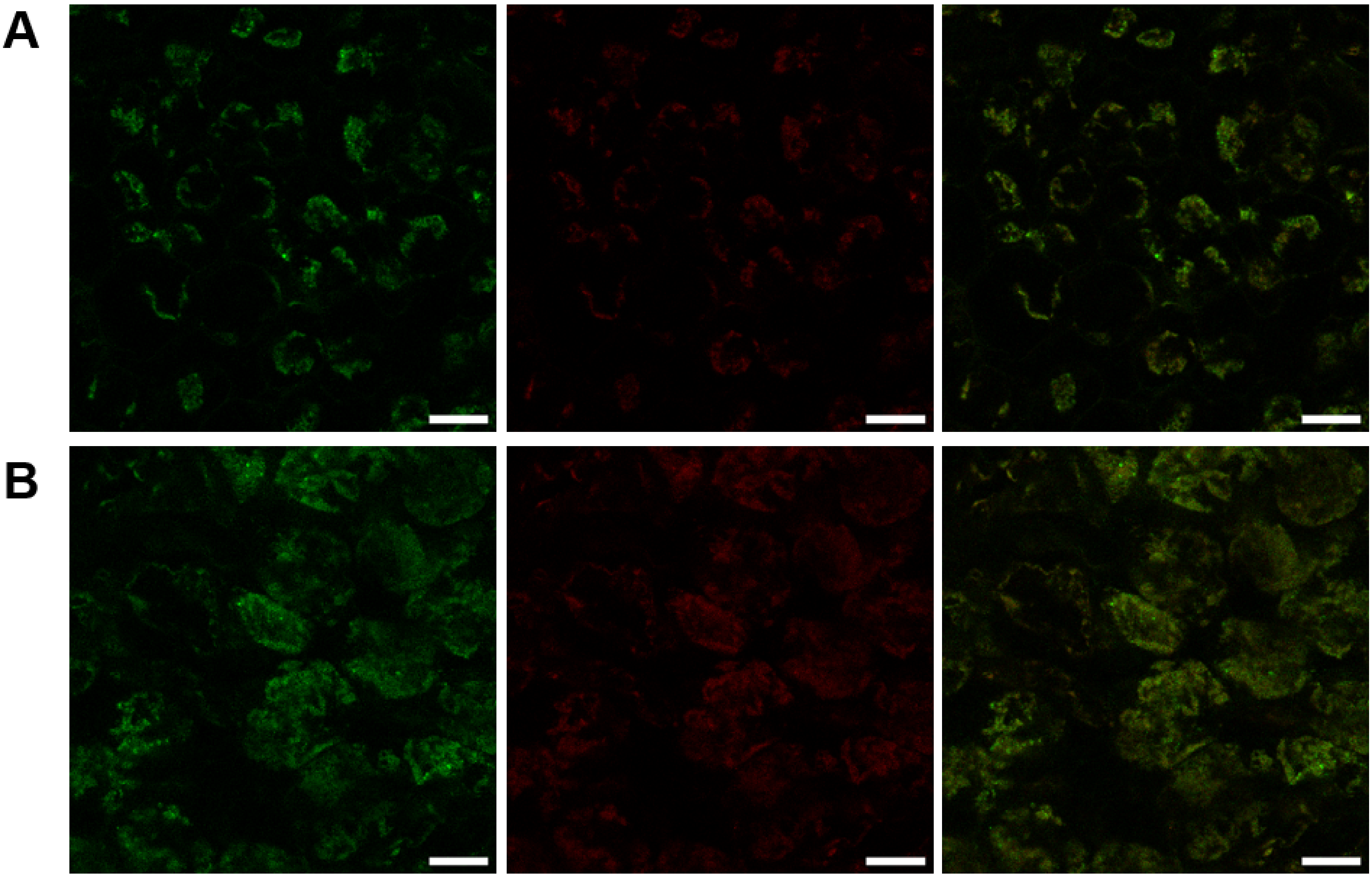

Figure S8. PGN recognizing proteins in *atmurE-3* seedlings. (**A**,**B**) Fluorescence microscopic image of *Arabidopsis atmurE-3* cells expressing (**A**) *DmPGRP$_{ΔSP}$-GFP* and (**B**) *HsPGRP$_{ΔSP}$-GFP* (green) together with a plastid marker (magenta; scale bars 20 µm).

Table S1. List of primers used in this study.

name	sequence
ddl1-S1	5′-CAGTTTCATGGCATTGGTGATC-3′
ddl1.3 End	5′-ACTTACATCTACATATCTTTGC-3′
ddl1.3 Start	5′-CACCATGGCGTCCATGGCGAC-3′
Ddl-A1	5′-CAAGTAGTTTGGTACTGTCATG-3′
SAIL-LB2	5′-GCTTCCTATTATATCTTCCCAAATTACCAATACA-3′
SALK-LB1	5′-AATCAGCTGTTGCCCGTCTCACTGGTGAA-3′
murE-S1	5′- CACTGCTTGCTTTCTCAGTCTC-3′
murE5-S2	5′- CATGGAAAACACCAAAGCCGATG- 3′
murE-A2	5′- CAACAGCACAACGTACAGCTAC -3′
Wisc-LB4	5′- TGATCCATGTAGATTTCCCGGACATGAAG-3′
murE-S3	5′- GGAAGGTAACACTAGGATCAAC-3′
murE-A3	5′- CGCATTGTCGAAATCCACTTC-3′
Wisc-LT6	5′- AATAGCCTTTACTTGAGTTGGCGTAAAAG-3′
DmPGRP-S2	5′- CACCATGGGAAAGTCTAGACAAAGATCTC-3′
DmPGRP-End	5′- AGGATTAGAAAGCCAATGAGG-3′
HsPGLYRP-S2	5′- CACCATGCAAGAAACTGAAGATCCTGCTTG-3′
HsPGLYRP-End	5′- AGGAGATCTATAATGAGGCCA-3′
AmiC1-End	5′- TCGTTTGAGGTACTGAAGAATACCGCGAG-3′
AmiC2-Start	5′- CACCATGGGGAAATTACTAGTTGTGATTGAC-3′
AmiC2-End	5′- ACGCTGTAAGTATTTTAGGATG-3′
AmiC1-Start	5′- CACCATGTCAGTATTCATCGACCCCGGACAC-3′

EcAmiC-AMIN-Start	5′- CACCATGCAGGTCGTGGCGGTGCGC-3′
EcAmiC-AMIN-End	5′- GGCCGGATAGAGGTCCATCACC-3′
